# Impact of statewide safe sleep legislation on hospital practices and rates of sudden unexpected infant deaths

**DOI:** 10.1186/s40621-020-00247-0

**Published:** 2020-06-12

**Authors:** Kirsten Bechtel, Marcie Gawel, Gregory A. Vincent, Pina Violano

**Affiliations:** 1 Department of Pediatrics, Section of Pediatric Emergency Medicine, Yale School of Medicine, New Haven, CT USA; 2 Department of Injury Prevention, Community Outreach, and Research, Yale New Haven Hospital, New Haven, CT USA; 3 Child Fatality Review Panel, New Haven, CT USA; 4 Office of the Chief Medical Examiner, New Haven, CT USA

**Keywords:** Sudden unexpected infant death (SUID), Legislation, Hospital practices, Positional asphyxia

## Abstract

**Background:**

Sudden Unexpected Infant Death (SUID) is the leading cause of death in the post-neonatal period in the United States. In 2015, Connecticut (CT) passed legislation to reduce the number of SUIDs from hazardous sleep environments requiring birthing hospitals/centers provide anticipatory guidance on safe sleep to newborn caregivers before discharge. The objective of our study was to understand the barriers and facilitators for compliance with the safe sleep legislation by birthing hospitals and to determine the effect of this legislation on SUIDs associated with unsafe sleep environments.

**Methods:**

We surveyed the directors and/or educators of the 27 birthing hospitals & one birthing center in CT, about the following: 1) methods of anticipatory guidance given to parents at newborn hospital discharge; 2) knowledge about the legislation; and 3) barriers and facilitators to complying with the law. We used a voluntary online, anonymous survey. In addition, we evaluated the proportion of SUID cases presented at the CT Child Fatality Review Panel as a result of unsafe sleep environments before (2011–2015) and after implementation of the legislation (2016–2018). Chi-Square and Fisher’s exact tests were used to evaluate the proportion of deaths due to Positional Asphyxia/Accident occurring before and after legislation implementation.

**Results:**

All 27 birthing hospitals and the one birthing center in CT responded to the request for the method of anticipatory guidance provided to caregivers. All hospitals reported providing anticipatory guidance; the birthing center did not provide any anticipatory guidance. The materials provided by 26/27 (96%) of hospitals was consistent with the American Academy of Pediatrics (AAP) Guidelines. There was no significant change in rates of SUID in CT before (58.86/100,000) and after (55.92/100,000) the passage of the legislation (*p* = 0.78). However, more infants died from positional asphyxia after (20, 27.0%) than before the enactment of the law (*p* < 0.01).

**Conclusions:**

Despite most CT hospitals providing caregivers with anticipatory guidance on safe sleep at newborn hospital discharge, SUIDs rates associated with positional asphyxia increased in CT after the passage of the legislation. The role of legislation for reducing the number of SUIDs from hazardous sleep environments should be reconsidered.

## Background

Sudden Unexpected Infant Death (SUID) is the leading cause of death in the post-neonatal period. An unsafe sleep environment often contributes to these deaths (Bass et al. 2018). On October 1, 2015, legislation was implemented in CT to reduce the number of SUIDs (*Connecticut General Statute 19a-55b – Information on newborn infant safe sleep practices*) (Connecticut General Statutes 19a-55b n.d.). This law requires, “each hospital, as defined in section 19a-490, through its maternity program, shall provide the parent or parents or the legal guardian of a newborn infant with written informational materials containing the most recent American Academy of Pediatrics’ (AAP) (2011) (Task force on Task Force on Sudden Infant Death Syndrome 2011) recommendations at the time legislation was passed concerning safe sleep practices at the time of such infant’s discharge from the hospital.” (Connecticut General Statutes 19a-55b n.d.) The informational materials should state that infants should sleep in the same bedroom as their parents – but on a separate surface (i.e., crib or bassinet) and for infants to share their parents’ bedroom for at least the first 6 months of life to decrease the likelihood of SUIDs (Task force on Task Force on Sudden Infant Death Syndrome 2011). Currently, there is no penalty for hospitals or birthing centers if they do not comply with this legislation. This legislation was passed in response to a report from the CT Child Fatality Review Panel and the CT Child Advocate that reported that sleep related deaths, due to unsafe sleeping environments, were the leading cause of deaths of healthy infants in CT (Office of the Child Advocate, State of Connecticut n.d.).

At present, only Florida, Illinois, and Michigan have legislation in accordance with previously published guidelines mandating birthing hospitals and centers provide anticipatory guidance to parents and caregivers regarding a safe sleep environment following an infant’s birth (National Council of State Legislatures 2019). Florida and Illinois are consistent with the National Institutes of Health (NIH) guidelines, and Michigan’s law is consistent with Safe Sleep Child Care Licensing Laws.

To date, there has been no evaluation of the effectiveness of such legislation. Therefore, we sought to understand the barriers and facilitators for compliance with CT legislation by birthing hospitals. We also sought to determine if the number of SUID’s associated with unsafe sleep environments, such as from positional asphyxia, declined after the implementation of this law.

## Methods

This was a cross-sectional survey study approved by the Human Research Protection Program (HRPP) of Yale School of Medicine. In June 2018, we contacted the medical directors, nursing directors, and/or educators of the 27 birthing hospitals and one birthing center in CT by phone to determine what infant safe sleep materials, if any, were provided as required by CT Law and to obtain permission to send a follow up email to voluntarily complete an online, anonymous survey to evaluate knowledge and implementation of the legislation. We asked them for the following information: 1) whether or not they were aware of CT’s legislation on safe sleep; 2) whether or not they were aware of any anticipatory guidance given to parents and/or caregivers regarding a safe sleep environment at newborn hospital discharge (e.g. video, written materials, verbal instruction); and 3) whether there were any barriers and/or facilitators to assist in complying with the legislation. Survey questions can be found in Table 1. We also asked these respondents to send their written safe sleep anticipatory guidance materials (electronically or via mail) to the study investigators. Materials received from the 27 birthing hospitals and the one birthing center were reviewed independently by three of the authors (KB, MG, PV) for their content to examine for consistency with AAP guidelines.

**Table 1:** Survey responses for the 27 birthing hospitals and one birthing centers

Survey question	Survey response (*n* = 14/27, 51.9%)	
Q1 - How many newborns are born at your hospital each year?	Mean = 1439 births/year (ME 411.05 95% CI)	
Q2 - Are you aware that there is a law in Connecticut (Public Act 15–39: An Act Concerning Safe Sleep Practices) that requires birthing hospitals/centers to provide written informational material on providing an infant with a safe sleep environment at hospital discharge?	Yes	12 (85.7%)
	No	2 (14.3%)
Q3 - At hospital discharge, who provides written informational material on safe sleep?	Physician	2 (11.11%)
	Advanced Practice Provider	1 (5.6%)
	Nurse	12 (66.7%)
	Obstetricians	3 (16.7%)
Q4 - In what languages are these written informational materials?	English	12 (50.0%)
	Spanish	10 41.7%)
	Language specific interpreter	1 (4.2%)
	Other	1 (4.2%)
Q5 - Does your hospital offer any additional resources to families to promote a safe sleeping environment, such as cribs?	Yes	3 (21.4%)
	No	8 (57.1%)
	Unsure	3 (21.4%)
Q6 - Describe any barriers you may encounter to deliver safe sleep informational materials to parents/caregivers of newborns at hospital discharge.	Language other than English.	8 (57.1%)
	Parent exhaustion/overwhelmed	6 (42.9%)
Q7 - Describe any facilitators that help you to deliver safe sleep informational materials to parents/caregivers of newborns at hospital discharge.	Free materials	11 (78.6%)
	Available in another language	3 (21.4%)
Q8 - If evidence-based, informational materials for safe sleep were provided free of charge, would you use them?	Yes	14 (100%)
	No	0 (0%)

### SUID rates

Next, we obtained data on CT SUID deaths from infant death records reviewed by the State of CT Child Fatality Review Panel (CFRP). We included infants up to 12 months old with deaths occurring from January 1, 2011 through December 31, 2018 for this study. Connecticut established the CFRP to review unexplained or unexpected circumstances of the death of any child less than 18 years old, regardless of involvement in a state department or agency addressing child welfare, social or human services, or juvenile justice. Currently, the CFRP meets monthly at the Office of the Chief Medical Examiner and reviews all deaths, regardless of involvement with state agencies, of children up to age 18 years old reported to the Office of the Chief Medical Examiner. Although the CFRP is an independent entity, its day-to-day operations are coordinated through the state’s Office of the Child Advocate.

The established CFRP is composed of thirteen voluntary permanent members (or their designee) as follows: the Child Advocate; the Commissioners of Children and Families, Public Health and Public Safety; the Chief Medical Examiner; the Chief State’s Attorney; a pediatrician, appointed by the Governor; a representative of law enforcement, appointed by the President Pro Tempore of the Senate; an attorney, appointed by the Majority leader of the Senate; a social work professional, appointed by the Minority Leader of the Senate; a representative of a community service group appointed by the Speaker of the House of Representatives; a psychologist, appointed by the Majority Leader of the House of Representatives; and an injury prevention representative, appointed by the Minority Leader of the House of Representatives. In addition, the CFRP, reflects the ethnic, cultural and geographic diversity of the state.

The following procedures are used by the CT Chief Medical Examiner in the determination of the cause and manner of death (National Association of Medical Examiners’ Panel on Sudden Unexpected Death in Pediatrics 2019). The forensic examination of a pediatric death must include the following components:

1. A thorough death scene investigation, ideally with a re-enactment of when the child was last seen alive and when the child was found unresponsive. Investigations and re-enactments must involve the people who were with the child at those times and should take place regardless of where or when the child was pronounced (i.e. if emergency medical services (EMS) obtains a return of circulation and the child dies in hospital a few days after being found, a re-enactment should still be performed).
2. A complete autopsy with histology, toxicology, and metabolic testing
3. Review of medical records

The following paragraphs describe the decision process for common infant cause of death determinations:

- Sudden infant death syndrome (SIDS): SIDS is a diagnosis of exclusion, and strict parameters must be set in order to make this determination. In order to classify a death as SIDS, the death must have occurred between 1 month and 1 year of age. The infant must have no pre-existing conditions, including pre-maturity. A complete scene investigation with a re-enactment show that the infant was found supine on an appropriate sleep surface. A complete autopsy must reveal no trauma or natural disease and radiology, histology, toxicology, and metabolic studies must be negative. The manner of death is natural in these cases. If any one of these criteria are not met, the diagnosis of SIDS cannot be made.
- Sudden unexpected death in infancy (SUID): SUID is a diagnosis used when a complete autopsy fails to reveal an adequate cause of death, but one or more of the criteria to diagnose SIDS are not met. If an autopsy is negative and the child is reported to have been found on an appropriate sleep surface, but a re-enactment was not performed, a diagnosis of SIDS cannot be made, and the diagnosis of SUID will be used. If scene investigation and re-enactment reveal that the infant was in an unsafe sleep environment in the supine position, and the autopsy does not show evidence of airway obstruction and is negative for trauma, natural disease, or intoxicants, the diagnosis of SUID can be made, and the manner of death is undetermined.
- Positional asphyxia: When there is clear evidence from the autopsy examination, scene investigation, and re-enactment of airway obstruction or restriction of breathing mechanics due to outside forces (chest compression) in the absence of natural disease, the diagnosis of positional asphyxia may be made. The manner of death in these cases is either accident or homicide, and that determination is based on the circumstances revealed in the scene investigation (National Association of Medical Examiners’ Panel on Sudden Unexpected Death in Pediatrics 2019).

### Statistical analysis

For the anticipatory guidance materials, results were compared, and inter-observer validity was calculated with the Kappa statistic for author agreement regarding material consistency with the AAP Recommendations. Those SUIDs where the infant was born in a CT birthing hospital/center and were determined to have the “Cause of Death as Positional Asphyxia and Manner of Death as an Accident,” as determined by the Connecticut Chief Medical Examiner, and occurred in the sleep environment were categorized as deaths that occurred in an unsafe sleeping environment. Rates were calculated using the population of infants through 12 months old in CT (State of Connecticut Department of Public Health n.d.). Chi-Square and Fisher’s exact was used to evaluate the proportion of deaths due to Positional Asphyxia/Accident that happened before (2011–2015) and after (2016–2018) the implementation of the legislation. SPSS Version 26 for Mac was used for statistical analysis. SPSS Version 26 [IBM Corp. Released 2018. IBM SPSS Statistics for Mac, Version 26.0. Armonk, NY: IBM Corp.].

## Results

All 27 birthing hospitals and the one birthing center responded to the request for the method of anticipatory guidance provided to parents and caregivers. The average census for newborn deliveries in these facilities was 1439 births/year. The single freestanding birthing center did not provide the parents and caregivers with any safe sleep anticipatory guidance. All 27 birthing hospitals provided anticipatory guidance. Upon review of the anticipatory guidance materials, 26/27 (96.3%) offered anticipatory guidance consistent with the 2011 American Academy of Pediatrics Guideline (Task force on Task Force on Sudden Infant Death Syndrome 2011). Of the 27 hospitals, only one hospital did not provide the advice to refrain from bed sharing. There was strong inter-observer agreement amongst authors for review of the materials (Kappa = .98).

Only 51.8% (14/27) of hospitals responded to the online survey. Of the respondents, 92.8% (13/14) stated that they knew about the legislation; 71.4% (10/14) reported their institutions provided materials in both English and Spanish; and 33.3% (4/14) provided parents and/or caregivers with supplies (e.g., sleep sacks, pack and plays, sleep boxes). Of the 14 respondents, 78.6% (11) reported free materials were a facilitator; 100% (14) said they would prefer to use free materials if available; and 57.1% (8) said providing anticipatory guidance to non-English speaking parents and/or caregivers was a barrier (Table 1).

Overall, there was no significant change in rates of SUID in CT before (58.86/100,000) and after (55.92/100,000) the passage of the legislation (*p* = 0.78) (CT Department of Health, personal communication). However, of significance, more infants died from positional asphyxia after (20, 27.0%) than before the enactment of the law (12, 10.8%) (*p* < 0.01) (Table 1). Additionally, fewer infants died from SIDS (*p* < 0.01) and from SUID (*p* = 0.05) (Table 2).

**Table 2:** Proportion of SUIDs based on cause and manner of death pre and post implementation of legislation

Cause of death/Manner of death	Pre legislation	Post legislation	*P*-value
SUID/UNDETERMINED	54 (48.6%)	25 (33.8%)	< 0.05
SIDS/NATURAL	18 (16.2%)	2 (2.7%)	< 0.01
POSTITONAL ASPHYXIA/ACCIDENT	12 (10.8%)	20 (27.0%)	< 0.01
UNDETERMINED/UNDETERMINED	27 (24.3%)	27 (36.5%)	0.11
TOTAL	111	74	

## Discussion

Our survey of the 27 birthing hospitals and the one birthing center found all the hospitals provided anticipatory guidance on safe sleep to parents and/or caregivers of newborns prior to discharge. No survey data were obtained from the one birthing center. Even though most CT hospitals complied with the safe sleep legislation, there was an increase in SUIDs due to positional asphyxia in CT after the passage of the legislation. To our knowledge, this is the first study to examine the association of SUID rates with respect to the passage of statewide safe sleep legislation. The role of statewide legislation, regarding the provision of safe sleep anticipatory guidance at newborn hospital discharge, to prevent SUIDs from positional asphyxia should be further examined.

The role of legislation in promoting safe child-care practices of parents and/or caregivers is somewhat mixed depending on the mechanism of injury. For child passenger safety, legislation to behoove parents and caregivers to use safe child caregiving practices for motor vehicle safety has been shown to change caregiver behavior and reduce injury. It has been well documented that legislation mandating the correct use of booster seats are effective in preventing motor vehicle related injuries and death (Mannix et al. 2012; Brubacher et al. 2016). However, legislation requiring daycare providers to provide a safe sleep environment for infants younger than 12 months old has been shown to not change the practice of daycare providers placing infants safely to sleep when observed in practice (Staton et al. 2019). Other authors have also suggested similar safe sleep legislation can work to reduce sleep-related deaths from hazardous sleeping conditions if there is a feedback loop between Child Death Review Panels and hospital providers to refine provider practices in modeling a safe sleep environment in the hospital for parents and caregivers (Moon et al. 2016; Krugman and Cumpsty-Fowler 2018).

There are many reasons why a hospital stay following an infant’s birth may not be the optimal time to provide parents and caregivers with anticipatory guidance regarding a safe sleep environment. First, the race or culture of the health care provider, such as a physician, may impact the trust parents and/or caregivers of the same race or culture have for the delivery and acceptance of anticipatory guidance (Hwang et al. 2017). Second, hospital facilities and staff may not always model recommended safe sleep practices, and this may contribute to the lack of adherence to safe sleep practices by parents and other parents and caregivers, despite the provision of anticipatory guidance (Kellams et al. 2017). Third, there may be hospital provider bias. For example, if providers do not agree with the AAP recommendations, they are unlikely to convey that they are safe and necessary to parents and/or caregivers (Colson et al. 2019). Finally, even if they do agree with AAP recommendations, hospital providers have reported limited ability to provide guidance on a safe sleeping environment because of the lack of time, due to the competing tasks of patient care and documentation in the medical record (Naugler and DiCarlo 2018).

It may be that additional content is necessary to overcome barriers to caregiver adherence to the AAP recommendations. For example, Colson et al. found in their qualitative study of hospital providers and safe sleep practices that several participants suggested personal stories from families whose infants died from sleep-related deaths could be shared to promote caregiver adherence (Colson et al. 2019). Additionally, it has been demonstrated that both parents and/or caregivers and providers may be reluctant to place infants in the supine position due to the concern of the infant choking (Naugler and DiCarlo 2018; Moon et al. 2010). Anticipatory guidance addressing this concern may aid the caregiver understanding and adherence with safe sleep guidelines.

Repeated guidance from multiple health care providers during the first year of life, after newborn hospital discharge, is also likely needed to reinforce safe sleep messaging and facilitate caregiver adherence (Von Kohorn et al. 2010). One study by Moon et al. demonstrated mobile text messages led to better caregiver adherence to safe sleep guidelines than did anticipatory nursing guidance at the time of newborn hospital discharge (Moon et al. 2017a). A second study by Moon et al. revealed participants who were contacted by text message were more likely to view a video and respond to queries about safe sleep than those reached by email (Moon et al. 2017b). Given the prevalence of cell phones, the use of either text message or apps may be an alternative method/mode for parents and caregivers to seek help at home when they struggle with providing the safest sleeping environment for their infants. This may be more impactful than mandated anticipatory guidance at newborn hospital discharge.

Should providing mandated newborn hospital discharge guidance on safe sleep to parents and caregivers cease? It is important to remember that, at least in CT, the content provided is consistent with the AAP recommendations for safe sleep (Task force on Task Force on Sudden Infant Death Syndrome 2011). A future study of caregivers of newborns delivered at a birthing hospital or center may be beneficial (asking if they ever received any materials and if so, how what the education delivered). One institution mentioned providers used a “teach-back” method, a means by which the understanding of parents and caregivers is assessed by having the parent/caregiver “teach” the provider how to provide a safe sleep environment for the newborn. The teach-back method has been shown to improve comprehension and retention of discharge instructions in the emergency department (Slater et al. 2017; Samuels-Kalow et al. 2016), even in a busy clinical setting (Griffey et al. 2015). Further research is needed to test the utility and feasibility of teach-back for routine use, including its impacts on future outcomes, such as SUIDs due to positional asphyxia.

Perhaps it may be helpful to explore the perspectives of community pediatricians as to their role in continuing to provide anticipatory guidance consistent with the AAP Recommendations. Schaeffer and Asnes, however, demonstrated in a qualitative evaluation of interviewed pediatricians that they are ambivalent about giving guidance regarding bed-sharing consistent with the AAP guideline (Schaeffer and Asnes 2018). Thus, it is unclear whether efforts including legislation to mandate delivery of guidance on safe sleep during well infant visits consistent with the AAP guidelines would also be useful.

There are limitations to our study. The first is only 52% of hospital providers completed the on-line survey, thus there may be responder bias. There was, however, 100% compliance with providing materials for the authors to review for consistency with the AAP guidelines, and therefore implied compliance with the legislation. Second, we did not survey the methods by which providers dispersed guidance at the time of discharge (i.e., was there a discussion or some assurance that the parents and/or caregivers understood and agreed with the given advice; or perhaps the educational materials were just handed to caregivers and were never read by the caregivers). Third, even though there were more deaths due to positional asphyxia, the proportion of caregivers putting their infants to sleep in a safe sleep environment before and after implementation of the law is unknown. In addition, there is the possibility of a diagnostic shift in the way the cause and manner of death were determined by the medical examiner during the period of the study. This may have resulted in more infants being classified as positional asphyxia and accident after passage of the legislation. Even if this is the case, the impact of the law would be negligible at best, since positional asphyxia is due to an unsafe sleep environment, the scenario to which the legislation was primarily addressing. As this is an ecological study causation of the legislation on SUIDs cannot be determined. True association also cannot be determined as we did not account for other factors with a multivariable analysis.

## Conclusion

In 2018, most CT hospitals provided parents and caregivers with printed anticipatory guidance on safe sleep at newborn hospital discharge consistent with AAP guidelines. However, rates of SUIDs associated with positional asphyxia in the sleeping environment increased in Connecticut after the safe sleep legislation was passed in 2015. The role of statewide legislation in the effectiveness of providing printed safe sleep anticipatory guidance at newborn hospital discharge to prevent SUIDs from unsafe sleep conditions should be reexamined. A future study to confirm these findings in other states with similar legislation is needed.

## Data Availability

Made upon request to authors.

## Abbreviations

AAP: American Academy of Pediatrics
CFRP: Child Fatality Review Panel
CT: Connecticut
HRPP: Human research protections program
NIH: National Institutes of Health
SIDS: Sudden infant death
SUID: Sudden Unexpected Infant Death
U.S.: United States
